# Cycle-consistent adversarial networks improves generalizability of radiomics model in grading meningiomas on external validation

**DOI:** 10.1038/s41598-022-10956-9

**Published:** 2022-04-29

**Authors:** Yae Won Park, Seo Jeong Shin, Jihwan Eom, Heirim Lee, Seng Chan You, Sung Soo Ahn, Soo Mee Lim, Rae Woong Park, Seung-Koo Lee

**Affiliations:** 1 Department of Radiology and Research Institute of Radiological Science and Center for Clinical Imaging Data Science, Yonsei University College of Medicine, 50-1 Yonsei-ro, Seodaemun-gu, Seoul, Korea; 2 Department of Biomedical Sciences, Ajou University Graduate School of Medicine, Suwon, Republic of Korea; 3 Department of Computer Science, Yonsei University, Seoul, Korea; 4 Department of Biomedical Informatics, Ajou University School of Medicine, Suwon, Republic of Korea; 5 Office of Biostatistics, Ajou Research Institute for Innovative Medicine, Ajou University Medical Center, Suwon, Republic of Korea; 6 Department of Biomedical Systems Informatics, Yonsei University College of Medicine, 50-1 Yonsei-ro, Seodaemun-gu, Seoul, Korea; 7 Department of Radiology, Ewha Womans University College of Medicine, Seoul, Korea

**Keywords:** Cancer imaging, CNS cancer, Machine learning, Computational biology and bioinformatics, Data processing

## Abstract

The heterogeneity of MRI is one of the major reasons for decreased performance of a radiomics model on external validation, limiting the model’s generalizability and clinical application. We aimed to establish a generalizable radiomics model to predict meningioma grade on external validation through leveraging Cycle-Consistent Adversarial Networks (CycleGAN). In this retrospective study, 257 patients with meningioma were included in the institutional training set. Radiomic features (*n* = 214) were extracted from T2-weighted (T2) and contrast-enhanced T1 (T1C) images. After radiomics feature selection, extreme gradient boosting classifiers were developed. The models were validated in the external validation set consisting of 61 patients with meningiomas. To reduce the gap in generalization associated with the inter-institutional heterogeneity of MRI, the smaller image set style of the external validation was translated into the larger image set style of the institutional training set using CycleGAN. On external validation before CycleGAN application, the performance of the combined T2 and T1C models showed an area under the curve (AUC), accuracy, and F1 score of 0.77 (95% confidence interval 0.63–0.91), 70.7%, and 0.54, respectively. After applying CycleGAN, the performance of the combined T2 and T1C models increased, with an AUC, accuracy, and F1 score of 0.83 (95% confidence interval 0.70–0.97), 73.2%, and 0.59, respectively. Quantitative metrics (by Fréchet Inception Distance) showed that CycleGAN can decrease inter-institutional image heterogeneity while preserving predictive information. In conclusion, leveraging CycleGAN may be helpful to increase the generalizability of a radiomics model in differentiating meningioma grade on external validation.

## Introduction

Meningiomas are the most common primary intracranial neoplasms in adults, accounting for approximately one-third of all intracranial tumors^1^. The majority of meningiomas (80%) are classified as low-grade (World Health Organization [WHO] grade 1; benign) and have an indolent clinical course^2^. On the other hand, high-grade (WHO grade 2 or 3; atypical or anaplastic) tumors have an aggressive biological behavior, a tendency to recur, and a poor prognosis^2^. The standard management typically involves surgical resection, and adjuvant radiation therapy is often recommended for high-grade meningiomas^3^. Therefore, developing a noninvasive generalizable model based on MRI to predict meningioma grade may assist clinical decision making by providing information on treatment planning, including surgical resection strategy^4^, and care of incidentally detected meningiomas in asymptomatic patients^3^.

MRI is the key imaging modality for diagnosis and characterization of meningioma and treatment decision^5^. Several studies applying radiomics, which translates radiological images into high-dimensional mineable imaging data^6^, have shown promising results in predicting meningioma grade^7–12^. However, majority of them did not perform external validation^7^. Those studies that performed external validation showed drastically decreased performance in external validation^10–12^, which limits the real-world application of radiomics models. Given that the objective of a prediction model is to predict outcomes in future patients, not to classify previously described characteristics, model generalizability on external validation is critical for model implementation^13^.

The inter-institutional heterogeneity of MRI protocol is a major reason for decreased performance of a radiomics model in the external validation stage^13^. Although consensus recommendations for standardized imaging protocol are established in brain tumors such as glioma or brain metastases^14,15^, consensus imaging protocol for meningiomas is currently lacking, which leads to substantial inter-institutional heterogeneity.

Recently, an approach based on the unpaired image-to-image translation using Cycle-Consistent Adversarial Networks (CycleGAN), a style transfer technique, has been suggested as a promising strategy to overcome poor model performance when dealing with external images^16^. CycleGAN can transfer the style of the image, while preserving the semantic information within the data^16^. The approaches using CycleGAN show superior visual similarities between image domains both quantitatively and qualitatively compared with other normalization methods and eliminate manual preparation of the representative reference image because they learn the whole image distribution^17,18^. We hypothesized that this approach can be applied to convert heterogeneous MRIs and lead to improved performance of a radiomics model to predict meningioma grade on external validation^17,18^. Thus, the objective of this study was to establish a generalizable radiomics model to predict meningioma grade on external validation through leveraging CycleGAN.

## Materials and methods

### Patient population

The Yonsei University Institutional Review Board approved this retrospective study and waived the need for obtaining informed patient consent. All methods were performed in accordance with the relevant guidelines and regulations. We identified 297 patients who were pathologically confirmed as having meningioma and underwent baseline conventional MRI between February 2008 and September 2018 in the institutional dataset. Patients with 1) missing MRI sequences or inadequate image quality (*n* = 17), 2) a previous history of surgery (*n* = 15), 3) a history of tumor embolization or gamma knife surgery before MRI exam (*n* = 5), and 4) an error in image processing (*n* = 2) were excluded. A total of 257 patients (low-grade, 162; high-grade, 95) were enrolled in the institutional cohort.

Identical inclusion and exclusion criteria were applied to identify 62 patients (low-grade, 47; high-grade, 15) from Ewha Mokdong University Hospital between January 2016 and December 2018 for external validation of the model. Patient flowchart is shown in Fig. S1.

### Pathological diagnosis

Pathological diagnosis was performed by neuropathologists, according to the WHO criteria^19^. The criteria for atypical meningioma (WHO grade 2) comprised 4–19 mitoses per 10 high-power fields, the presence of brain invasion, or the presence of at least three of the following features: “sheet-like” growth, hypercellularity, spontaneous necrosis, large and prominent nucleoli, and small cells. The criteria for anaplastic meningioma (WHO grade 3 comprised frank anaplasia (histology resembling carcinoma, sarcoma, or melanoma) or elevated mitoses (> 20 mitoses per 10 high-power fields)^19^.

### MRI protocol

In the institutional training dataset, patients were scanned on 3.0 Tesla MRI units (Achieva or Ingenia; Philips Medical Systems). Imaging protocols included T2-weighted (T2) and contrast-enhanced T1-weighted imaging (T1C). T1C images were acquired after administration of 0.1 mL/kg of gadolinium-based contrast material (Gadovist; Bayer).

In the external validation sets, patients were scanned on 1.5 or 3.0 Tesla MRI units (Avanto; Siemens, or Achieva; Philips Medical Systems), including T2 and T1C images. T1C images were acquired after administration of 0.1 mL/kg of gadolinium-based contrast material (Dotarem; Guerbert, or Gadovist; Bayer). Substantial variation existed between the acquisition parameters for T2 and T1C among the various MRI units between the institutional and external validation sets and reflected the heterogeneity of meningioma imaging data in clinical practice (Supplementary Table 1).

### Image preprocessing and radiomics feature extraction

Image resampling to 1-mm isovoxels, low-frequency intensity non-uniformity correction by the N4 bias algorithm, and co-registration of T2 images to T1C images were performed using Advanced Normalization Tools (ANTs)^20^. After skull stripping by Multi-cONtrast brain STRipping (MONSTR)^21^, signal intensities were z-score normalized. An affine registration was performed to transform the brain images to the MNI152^22^.

A neuroradiologist (with 9 years of experience) who was blinded to the clinical information semi-automatically segmented the entire tumor (including cystic or necrotic changes) on the T1C images using 3D Slicer software (v. 4.13.0; www.slicer.org) with edge- and threshold-based algorithms. Another neuroradiologist (with 16 years of experience) re-evaluated and confirmed the segmented lesions.

Radiomic features were calculated with a python-based module (PyRadiomics, version 2.0)^23^, with a bin size of 32. They included (1) 14 shape features, (2) 18 first-order features, and 3) 75 s-order features (including gray-level co-occurrence matrix, gray-level run-length matrix, gray-level size zone matrix, gray-level dependence matrix, and neighboring gray tone difference matrix) (Supplementary Material S1 and Supplementary Table 2). The features adhered to the standard sets by the Image Biomarker Standardization Initiative ^24^. A total of 214 radiomic features (107 × 2 sequences) were extracted.

### Radiomics model construction

**Figure 1:**
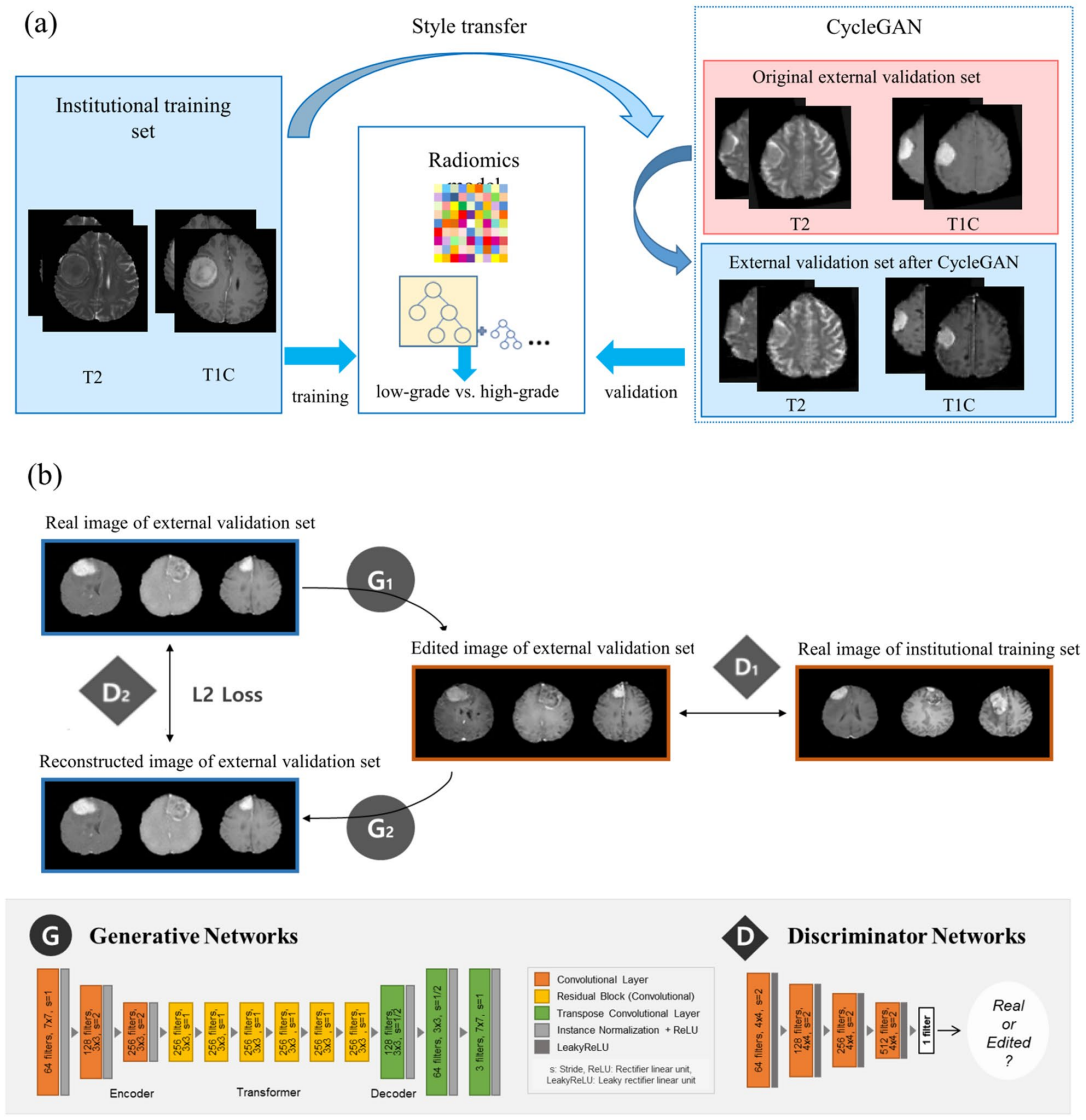
(**a**) Overall pipeline of the CycleGAN and radiomics for meningioma grading. (**b**) General network architecture of CycleGAN. CycleGAN = Cycle-Consistent Adversarial Networks, T1C = postcontrast T1-weighted image, T2 = T2-weighted image.

The schematic of radiomics model construction and establishment of an application system based on CycleGAN is shown in Fig. 1a. Radiomic features were MinMax normalized. Because the number of radiomic features was larger than the number of patients, mutual information was applied to select the significant features. The base radiomics classifiers were constructed using extreme gradient boosting with tenfold cross-validation in the training set. Synthetic minority over-sampling technique was applied for oversampling the minority class^25^. To improve the predictive performance and avoid potential overfitting, Bayesian optimization, which searched the hyperparameter space for optimal hyperparameter combinations, was applied. The area under the curve (AUC), accuracy, sensitivity, specificity, and F1 score (definitions shown in Supplementary Material S2) were obtained. Feature selection and machine learning process were performed using Python 3 with the Scikit-Learn library module (version 0.24.2).

### CycleGAN application

Figure 1b shows the general network architecture of CycleGAN. The generative adversarial network (GAN) has two neural networks, namely, a generator and a discriminator, for distinctive purposes. The CycleGAN uses two sets of GAN for style transfer to train unsupervised image translation models^16^. Unpaired institutional training and external validation datasets were used to train the discriminators and generators of CycleGAN.

To be delivered into CycleGAN^16^, the brain MRIs were converted to two-dimensional images in each aspect of the axial, sagittal, and coronal planes. Because the image size was diverse between institutions and individuals, the images were resized to 99 × 117 × 95 pixels after MNI152 template registration and to 116 × 116 pixels before putting them into CycleGAN.

In the first set of GAN, the first generator (G1) in CycleGAN converts the images from the external validation dataset to the domain of the institutional training dataset, while the first discriminator D1 checks if the images computed by G1 are real or fake (generated). Through this process, synthetic images from G1 become better with the feedback of their respective discriminators. In the second set of GAN, the second generator (G2) transfers the synthetic image generated from the first generator (G1) back to the original external validation dataset image, while the second discriminator (D2) checks if the images computed by G2 are real or fake (generated). Through this process, the trained CycleGAN model transferred the style of the external validation images to the training set. The cycle consistency loss, which is the difference between the generated output and the input image, was calculated and used to update the generator models in each training iteration^16^. L2 loss, which is known to accelerate the training process and generate sharp and realistic images in GAN^26,27^, was employed to estimate the cycle consistency loss. Inference results were randomly sampled and checked by a neuroradiologist (with 9 years of experience) for plausibility. External validation set images after CycleGAN were subjected to assess the performance of the radiomics model compared with the original external validation dataset. Because original external validation set and external validation set images after CycleGAN were independent from the radiomics modeling in the training process, there is no potential data breach^28^. Details of the CycleGAN architecture are shown in Supplementary Table 3.

### Evaluation of the effect of CycleGAN: Fréchet Inception Distance and t-Distributed Stochastic Neighbor Embedding

The Fréchet Inception Distance (FID) was calculated to measure the similarity between two datasets of images to measure the model quality quantitatively by evaluating the generated data (Supplementary Material S3)^29^. FID is an extension of the Inception Score^30^ and compares the distribution of generated images with the distribution of real images that were used to train the generator. FID has been shown to be consistent with human judgments and more robust to noise than inception score^29^. Three FID scores, namely, “training vs. original external validation,” “original external validation vs. transferred external validation,” and “training vs. transferred external validation” were calculated. To visualize the effect of CycleGAN on the extracted radiomic features, the high-dimensional feature space was projected and visualized into a lower dimensional space by using a two-dimensional t-Distributed Stochastic Neighbor Embedding (t-SNE) manifold^31^.

## Results

Patient characteristics in the institutional training (*n* = 257) and external validation sets (*n* = 61) are summarized in Table 1. The proportion of female sex was higher in the high-grade meningiomas in the training set (*p* < 0.001), but not in the external validation set (*p* = 0.833). No significant differences were found in other clinical characteristics between the training and external validation sets.

**Table 1:** Patient characteristics in the institutional training and external validation sets.

Variables	Institutional training set (*n* = 257)			External validation set (*n* = 61)			*P*-value^✝^
	Low-grade (*n* = 162)	High-grade (*n* = 95)	*P*-value*	Low-grade (*n* = 46)	High-grade (*n* = 15)	*P*-value*	
**Clinical**							
Age (years)	56.44 ± 12.08	58.40 ± 14.01	0.226	55.13 ± 13.61	56.73 ± 19.25	0.723	0.387
Female sex	138 (85.2)	59 (62.1)	< 0.001	31 (67.4)	10 (66.7)	0.959	0.127
Skull base location	31 (19.1)	26 (27.4)	0.125	12 (26.1)	5 (33.3)	0.587	0.344

Data are expressed as mean with standard deviation in parentheses or number with percentage in parentheses.

*Calculated from Student’s *t*-test for continuous variables and Chi-square test for categorical variables to compare the characteristics between low-grade and high-grade patients of the institutional cohort and the external validation set.

✝Calculated from Student’s *t*-test for continuous variables and Chi-square test for categorical variables for comparison of institutional training and external validation sets.

### Performance of the classifier for the original external validation and CycleGAN style-transferred external validation images

A total of 27 radiomic features were identified to differentiate meningioma grade (10 features from T2 and 17 features from T1C; 6 shape features, 2 first-order features, and 19 s-order features; details on Supplementary Table 4 and Fig. S2). In the institutional training set, the best performing classifier was achieved in the combined T1C and T2 models, with an AUC, accuracy, sensitivity, specificity, and F1 score of 0.88 (95% confidence interval [CI] 0.77–0.87), 77.7%, 82.8%, and 72.6%, respectively. The T2 and T1C models showed lower performances, with AUCs of 0.84 (95% CI 0.79–0.89) and 0.85 (95% CI 0.82–0.88), respectively.

In the external validation dataset before CycleGAN application, the T2 model showed the highest performance, with an AUC, accuracy, sensitivity, specificity, and F1 score of 0.78 (95% CI 0.64–0.92), 62.7%, 92.3%, 54,4%, and 0.52, respectively. The combined T2 and T1C models showed a similar performance, with an AUC of 0.77 (95% CI 0.63–0.91), whereas the T1C model showed the lowest performance, with an AUC of 0.73 (95% CI 0.58–0.87), respectively.

**Figure 2:**
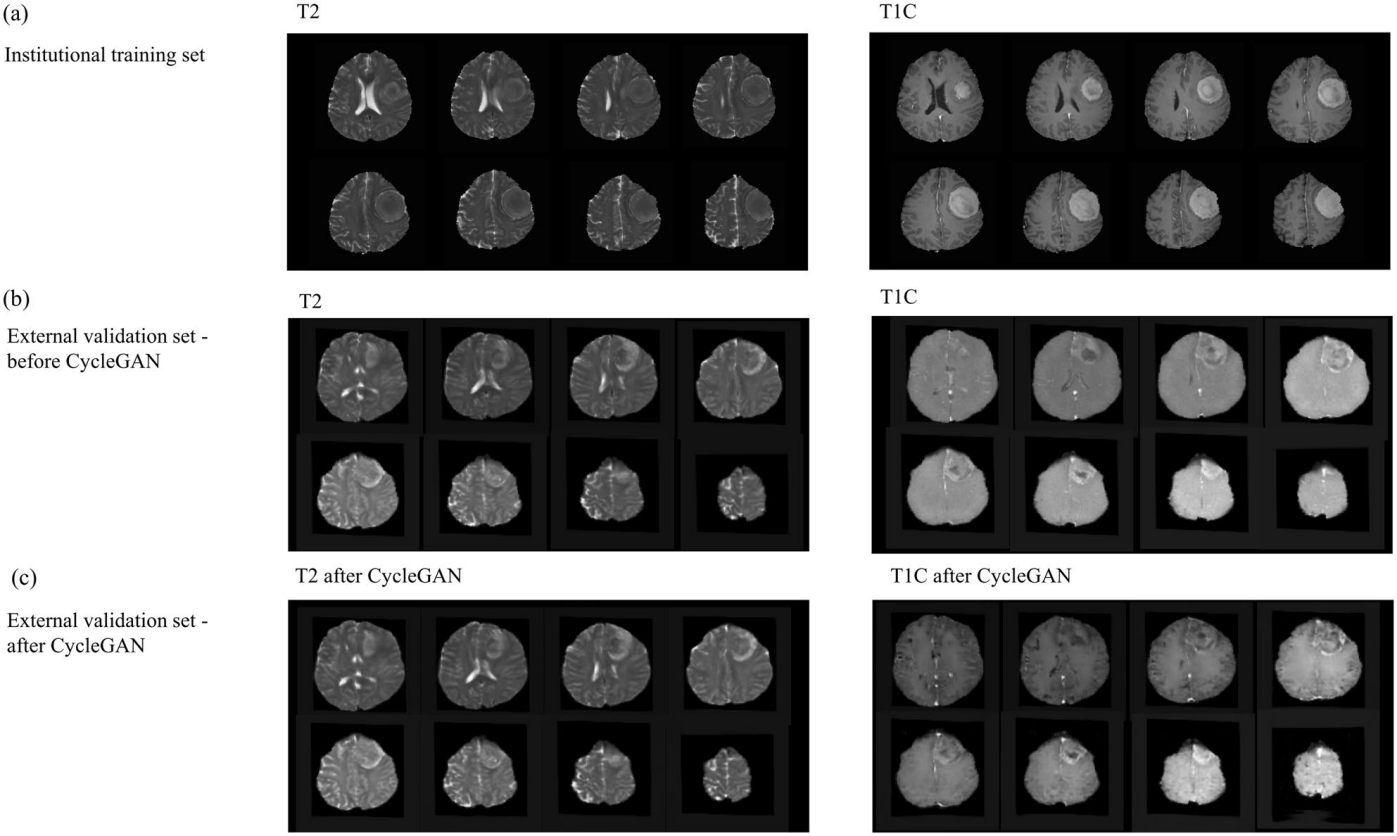
Representative cases showing the institutional training dataset and images before and after style transfer of the external validation set. (**a**) T2 and T1C images of a patient from the institutional training dataset that were leveraged to generate the radiomics model. (**b**) T2 and T1C images from the original external validation. (**c**) Style of T2 and T1C images from the external validation set after transformation to match that of the institutional training set images by using CycleGAN. CycleGAN = Cycle-Consistent Adversarial Networks, T1C = postcontrast T1-weighted image, T2 = T2-weighted image.

**Figure 3:**
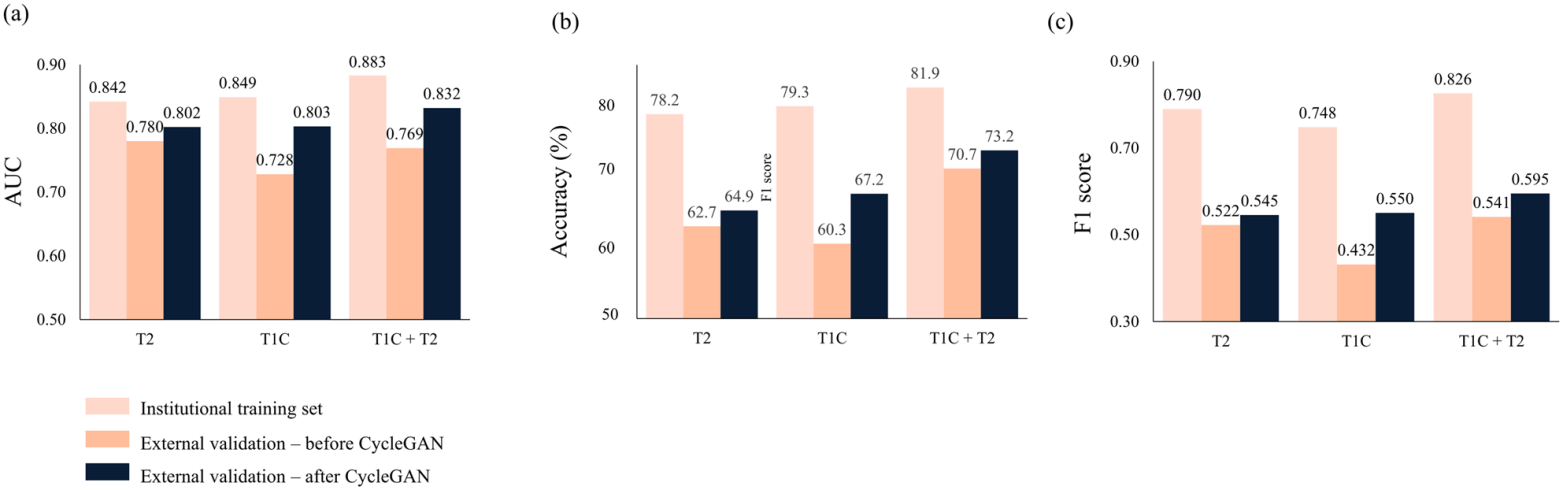
Radiomics model performance on the (**a**) AUCs, (**b**) accuracies, and (**b**) F1 scores of the radiomics model in the institutional training set and external validation set before and after applying CycleGAN. AUCs = areas under the curve, CycleGAN = Cycle-Consistent Adversarial Networks, T1C = postcontrast T1-weighted image, T2 = T2-weighted image.

**Figure 4:**
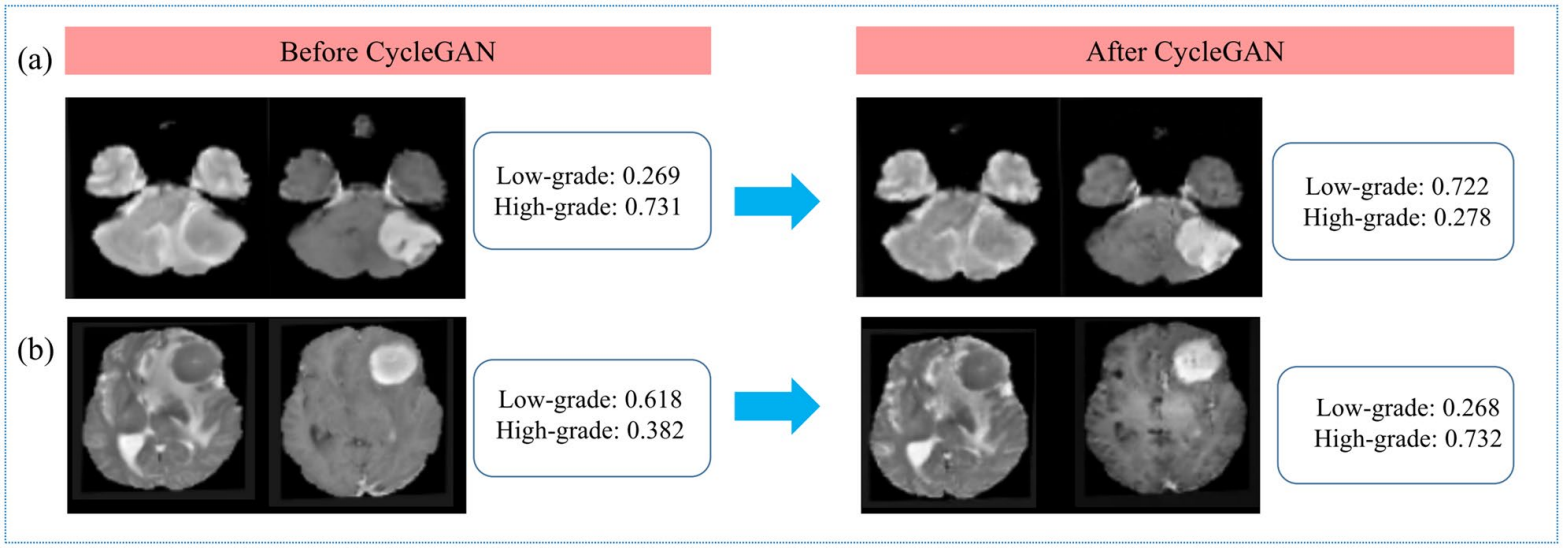
Representative cases showing the improvement in classifications after CycleGAN. Predictive scores close to 1.0 indicate that the model predicts the meningioma grade with confidence. (**a**) A case incorrectly diagnosed as high-grade meningioma before style transfer but correctly diagnosed as low-grade meningioma after style transfer. (**b**) A case incorrectly diagnosed as low-grade meningioma before style transfer but correctly diagnosed as high-grade meningioma after style transfer. CycleGAN = Cycle-Consistent Adversarial Networks.

The style of T2 and T1C images of the original external validation dataset was transformed to match that of the institutional training set images by using CycleGAN (Fig. 2). In the external validation dataset after CycleGAN application, the performance of all three radiomics models increased, although it did not reach statistical significance (Ps > 0.05). The combined T1C and T2 models showed the highest performance, with an AUC, accuracy, sensitivity, specificity, and F1 score of 0.83 (95% CI 0.70–0.97), 73.2%, 84.6%, 69.8%, and 0.59, respectively. T2 and T1C models showed lower performance, with AUCs of 0.80 (95% CI 0.66–0.95) and 0.80 (95% CI 0.67–0.93), respectively. The performance of the radiomics models in the institutional training set and external validation set before and after applying CycleGAN is shown in Table 2. Figure 3 shows the performance of the radiomics models, demonstrating the effect of style transfer by CycleGAN on the classification performance of the radiomics model on external validation. Figure 4 shows representative cases demonstrating the improvement in classifications after CycleGAN. Predictive scores close to 1.0 indicate that the model predicts the meningioma grade with confidence.

**Table 2:** Model performance on institutional training set and external validation set before and after applying CycleGAN.

	AUC (95% CI)	Accuracy (%)	Sensitivity (%)	Specificity (%)	F1 score
**Institutional training set**					
T2	0.84 (0.79–0.89)	78.2	82.9	73.5	0.79
T1C	0.85 (0.82–0.88)	79.3	83.4	75.2	0.75
T2 + T1C	0.88 (0.83–0.93)	81.9	85.1	78.9	0.83
**External validation set before applying CycleGAN**					
T2	0.78 (0.64–0.92)	62.7	92.3	54.4	0.52
T1C	0.73 (0.58–0.87)	60.3	61.5	60.0	0.43
T2 + T1C	0.77 (0.63–0.91)	70.7	76.9	68.9	0.54
**External validation set after applying CycleGAN**					
T2	0.80 (0.66–0.95)	64.9	92.3	56.8	0.55
T1C	0.80 (0.67–0.93)	67.2	76.9	64.4	0.55
T2 + T1C	0.83 (0.70–0.97)	73.2	84.6	69.8	0.59

AUC = area under the curve, CI = confidence interval, CycleGAN = Cycle-Consistent Adversarial Networks, T1C = postcontrast T1-weighted image, T2 = T2-weighted image.

### Evaluation of the effect of CycleGAN: FID and t-SNE

The FID scores by our proposed model are shown in Fig. S3. The FID score was highest in the “training vs. original external validation,” followed by the “training vs. transferred external validation” and then by the “original external validation vs. transferred external validation.” The FID score decreased to 52.2% after style transfer in the external validation set. However, the “original external validation vs. transferred external validation” showed the lowest FID score, which was 25.6% of the FID score of the “training vs. original external validation”.

The resulting extracted radiomics feature space on t-SNE for the institutional training and external validation sets before and after style transfer is shown in Fig. S4. Before the style transfer, a marked difference was noted between the institutional training and external validation test sets on the values of computed radiomic features. After applying CycleGAN, this influence was markedly decreased. The density plot shows that the distributions of the selected radiomic features of the external validation set became more similar to the institutional training set after applying CycleGAN (Fig. S5).

## Discussion

We demonstrated that leveraging CycleGAN is an effective approach to increase the generalizability of radiomics model on external validation. The areas under the curve of the combined T2 and T1C models in the external validation set increased from 0.77 to 0.83 after CycleGAN application. The FID score and t-SNE showed that data distributions between the institutional training and external validation sets in the image and radiomic feature levels became more similar after applying CycleGAN. To the best of our knowledge, this study is the first to investigate CycleGAN strategy for brain tumor imaging to develop a generalizable radiomics model.

CycleGANs are a relatively novel type of conditional generative adversarial networks, which have received considerable attention because of their ability to capture the characteristics of a single image collection and to generate synthetic images in the absence of any paired training examples^16,32^. Previous generative adversarial network studies on brain imaging have mainly focused on generating missing brain MRI data^33–35^ or creating high-resolution images from low-resolution images^36^, which requires ground truth sequences. However, no “ground truth” dataset of paired training examples (consisting of internal and external MRI examinations of identical patients at the same period) exists in real-world clinical practice. In our study, we focused on a practical and crucial problem encountered in implementing a machine learning model in medical imaging, which is increasing the generalizability of a radiomics model on external validation. External validation is a crucial process in models with artificial intelligence, because internal validation itself cannot guarantee model generalizability^13,37^. However, classical preprocessing steps, such as isovoxel resampling, bias field correction, and signal intensity normalization, are insufficient to counter image heterogeneity. We speculate that CycleGAN may be a practical approach to solve the image heterogeneity of an external dataset. A recent study has shown that CycleGAN can reduce the heterogeneity between radiomic features and increase reproducibility in chest radiographs, which is in line with our study^38^.

A notable finding in our study was that the T2 radiomics model showed relatively less decreased performance in the external validation set before applying CycleGAN, whereas the T1 radiomics model showed a larger decrease in performance in the external validation set before applying CycleGAN. Compared with the T1C protocols with different protocols, T2 protocols are relatively similar between institutions and less prone to failures from image acquisition artifacts^39^, which may lead to higher performance on external validation than the T1C model. Nonetheless, after CycleGAN application, the combined T2 and T1C models showed the highest performance. This finding suggests that CycleGAN may preserve the biological information from T2 and T1C sequences while effectively removing inter-institutional variation. Our results are in concordance with other studies that demonstrate that single sequence models have limited ability to reflect the underlying pathophysiology of meningiomas^9,40^.

Our external validation dataset included different scanner vendors, acquisition protocols, image reconstruction algorithms, and field strengths, resulting in large heterogeneity, which reflects the real-world clinical dataset in meningiomas^41^. Apart from the different MRI vendors with different field strengths, the resolution, sequence, echo time, repetition time, and inversion time have also not reached consensus in meningioma imaging. All of these differences induce heterogeneity of the MRI datasets, which poses as a unique challenge in the generalizability of the artificial intelligence in this area. Collecting heterogeneous labeled data from multiple institutions worldwide is the best solution to overcome this challenge. Nonetheless, even if we tackle this daunting challenge, the generalizability of the resulting artificial intelligence model cannot be fully guaranteed, as the data in another institution are possibly out-of-distribution. In this study, we demonstrate that leveraging an image harmonizing technique based on deep learning is feasible to increase generalizability in radiomics application for grading meningiomas.

The FID score was lowest in the “original external validation vs. transferred external validation,” rather than in the “training vs. transferred external validation” datasets. Considering the equation in FID^42^, which calculates the difference between the synthetic and real data distributions, the transferred external validation dataset has understandably the most close resemblance to the original external validation dataset. Nonetheless, the FID score from the “original external validation vs. transferred external validation” datasets decreased to 52.2% compared with that from the “training vs. original external validation” datasets. This result demonstrates that the data distributions between the training and external validation sets became more similar after applying CycleGAN.

This study has several limitations. First, it was conducted with a relatively small amount of data, particularly in the external validation set. As this is a technical feasibility study, a larger multi-institutional validation set is warranted to demonstrate significant performance improvement with CycleGAN. Second, we used two-dimensional CycleGAN rather than three-dimensional CycleGAN because of relative paucity of data. This may lead to slice-to-slice inconsistencies, which may adversely affect the performance. However, despite these limitations, as shortfall in generalization to real-world datasets with heterogeneous imaging data is the major barrier for the adoption of artificial intelligence in medical imaging, the strength of our study is that we demonstrated that CycleGAN is a feasible approach to tackle this challenging issue.

In conclusion, CycleGAN is potentially helpful in increasing the generalizability of a radiomics model in differentiating meningioma grade on external validation.

## Supplementary Information

Supplementary Information 1.

Supplementary Information 2.

Supplementary Information 3.

Supplementary Information 4.

Supplementary Information 5.

Supplementary Information 6.

## Abbreviations

AUC: Area under the curve
CycleGAN: Cycle-Consistent Adversarial Networks
FID: Fréchet Inception Distance
GAN: Generative adversarial network
T1C: Postcontrast T1-weighted image
T2: T2-weighted image
t-SNE: T-Distributed Stochastic Neighbor Embedding
WHO: World Health Organization
XGBoost: Extreme gradient boosting
