# Supplementary material for: Cycle-consistent adversarial networks improves generalizability of radiomics model in grading meningiomas on external validation
Source: Sci Rep. 2022 Apr 29;12:7042. doi: 10.1038/s41598-022-10956-9 (PMC9055063; doi:10.1038/s41598-022-10956-9)
Supplement: Supplementary file 1 — Supplementary Information 1. [file 41598_2022_10956_MOESM1_ESM.docx]

Supplementary data for:

**Cycle-Consistent Adversarial Networks Improves Generalizability of Radiomics Model in Grading Meningiomas on External Validation**

**Contents:**

S1. Radiomic features.

S2. Definition of F1 score

S3. Definition of Fréchet Inception distance (FID)

Supplementary Figure 1. Patient flowchart in the (a) institutional and (b) external validation datasets.

Supplementary Figure 2. MI scores of the selected radiomic features.

Supplementary Figure 3. Bar graph showing the FID score between the institutional training set and original external validation set, and the FID score between the institutional training set and external validation set after style transfer.

Supplementary Figure 4. t-SNE visualizations of radiomic feature vectors in the institutional training set and external validation set before and after style transfer with CycleGAN.

Supplementary Figure 5. Examples of the density plots showing the distribution of the selected radiomic features.

Supplementary Table 1. Comparison of MRI parameters of patients in the training cohort (from our institution) and those in the external validation cohort.

Supplementary Table 2. List of extracted radiomics features.

Supplementary Table 3. Detailed CycleGAN Architecture

Supplementary Table 4. List of significant multiparametric radiomic features to differentiate meningioma grade using MI.

**S1. Radiomic features**

Radiomic features were calculated with a python-based module (PyRadiomics, version 2.0) ^1^, with a bin size of 32, and included 1) 14 shape features, 2) 18 first order features, and 3) 75 second order features (including gray level co-occurrence matrix, gray level run-length matrix, gray level size zone matrix, gray level dependence matrix, and neighboring gray tone difference matrix). Shape features were extracted from the segmented mask, and first-order and second-order features were estimated using signal intensity and volume.

***1. Shape features***

These features compute the three-dimensional size and shape of volume of interest $V(x,y,z)$. Let $Vol$ and $A$ denote the volume and surface area, respectively.

| **Elongation** | $\sqrt{\frac{\lambda_{minor}}{\lambda_{major}}}$ | **Maximum 3D diameter** | The largest pairwise Euclidean distance between ROI surface mesh vertices |
| --- | --- | --- | --- |
| **Flatness** | $\sqrt{\frac{\lambda_{least}}{\lambda_{major}}}$ | **Mesh volume** | $V_{i}=\frac{O_{a_{i}}\cdot(O_{b_{i}}\times O_{c_{i}})}{6}$  $V=\sum_{i=1}^{N_{f}} V_{i}$ |
| **Least axis length** | $4\sqrt{\lambda_{least}}$ | **Minor axis length** | $4\sqrt{\lambda_{minor}}$ |
| **Major axis length** | 4$\sqrt{\lambda_{major}}$ | **Sphericity** | $\frac{\sqrt[3]{36\pi V^{2}}}{A}$ |
| **Maximum 2D diameter (column)** | The largest pairwise Euclidean distance between ROI surface mesh vertices in the row-column plane. | **Surface area** | $A_{i}=\frac{1}{2}\left\vert a_{i}b_{i}\times a_{i}c_{i} \right\vert$  $A=\sum_{i=1}^{N_{f}} A_{i}$ |
| **Maximum 2D diameter(row)** | The largest pairwise Euclidean distance between ROI surface mesh vertices in the column-slice plane. | **Surface area to volume ratio** | $\frac{A}{V}$ |
| **Maximum 2D diameter(slice)** | The largest pairwise Euclidean distance between ROI surface mesh vertices in the row-slice plane. | **Voxel volume** | $V_{voxel}=\sum_{k=1}^{N_{v}} V_{k}$ |

***2. First-order features***

First-order features describe the distribution of voxel intensities within the image region defined by the mask through commonly used and basic metrics.

X is a set of 𝑁𝑝 voxels included in the ROI. P(i) refers to the first-order histogram with 𝑁_𝑔_ discrete intensity levels, where 𝑁_𝑔_ is the number of non-zero bins, equally spaced from 0 with a width defined in the binWidth parameter and p(i) is the normalized first order histogram and equal to $\frac{P(i)}{N_{p}}$. 𝜖 is an arbitrarily small positive number (≈ 2.2 × 10^−16^).

| **Energy** | $\sum_{i=1}^{N_{p}} {(X\left( i \right)+c)}^{2}$ | **Interquartile Range** | $P_{75}{- P}_{25}$ |
| --- | --- | --- | --- |
| **Total energy** | $V_{voxel}\sum_{i=1}^{N_{p}} {(X\left( i \right)+c)}^{2}$ | **Range** | max(X)$-$ min(X) |
| **Entropy** | $-\sum_{i=1}^{N_{g}} p(i)\log_{2} (p\left( i \right)+ \epsilon)$ | **Mean absolute deviation (MAD)** | $\frac{1}{N_{p}}\sum_{i=1}^{N_{p}} \left\vert X\left( i \right)-\bar{X} \right\vert$ |
| **Minimum** | min($X$) | **Robust Mean Absolute Deviation**  **(rMAD)** | $\frac{1}{N_{10-90}}\sum_{i=1}^{N_{10-09}} \left\vert X_{10-90}\left( i \right)-\bar{X}_{10-90} \right\vert$ |
| **10^th^ percentile** | The 10^th^ percentile of $X$ | **Root mean squared (RMS)** | $\sqrt{\frac{1}{N_{p}}\sum_{i=1}^{N_{p}} {(X\left( i \right)+c)}^{2}}$ |
| **90^th^ percentile** | The 90^th^ percentile of $X$ | **Skewness** | $\frac{\frac{1}{N_{p}}\sum_{i=1}^{N_{p}} {(X\left( i \right)-\bar{X})}^{3}}{\left( \sqrt{\frac{1}{N_{p}}\sum_{i=1}^{N_{p}} {(X\left( i \right)-\bar{X})}^{2}} \right)^{3}}$ |
| **Maximum** | max($X$) | **Kurtosis** | $\frac{\frac{1}{N_{p}}\sum_{i=1}^{N_{p}} {(X\left( i \right)-\bar{X})}^{4}}{\left( \frac{1}{N_{p}}\sum_{i=1}^{N_{p}} {(X\left( i \right)-\bar{X})}^{2} \right)^{2}}$ |
| **Mean** | $\frac{1}{N_{p}}\sum_{i=1}^{N_{p}} X\left( i \right)$ | **Variance** | $\frac{1}{N_{p}}\sum_{i=1}^{N_{p}} {(X\left( i \right)-\bar{X})}^{2}$ |
| **Median** | The median gray-level intensity within the ROI. | **Uniformity** | $\sum_{i=1}^{N_{g}} {p(i)}^{2}$ |

***3. Second-order features***

Second-order features, widely used in pattern recognition, refer to higher order statistical measures and summarize the local spatial arrangement of intensities.

***3-1. Gray level co-occurrence matrix (GLCM) features***

Gray level co-occurrence matrix (GLCM) of size $N_{g}\times N_{g}$ described the second-order joint probability function of an image region constrained by the mask and was defined as $P(i, j|\delta, \theta)$. The ${(i, j)}^{th}$ element of this matrix represented the number of times the combination of levels $i$ and $j$ occurred in two pixels in the image, that were separated by a distance of δ pixels along angle θ. The distance δ from the center voxel was defined as the distance according to the infinity norm. For δ = 1, this resulted in 2 neighbors for each of 13 angles in 3D (26-connectivity), and a 98-connectivity for δ = 2.

Let ϵ be an arbitrarily small positive number (≈ 2.2 × 10^−16^), $P\left( i, j \right)$ be the co-occurrence matrix for an arbitrary δ and θ, $p\left( i, j \right)$ be the normalized co-occurrence matrix and equal to $\frac{P\left( i, j \right)}{\sum P\left( i, j \right)}$, $N_{g}$ be the number of discrete intensity levels in the image, $p_{x}\left( i \right)=\sum_{j=1}^{N_{g}} P\left( i, j \right)$  be the marginal row probabilities, $p_{x}\left( i \right)=\sum_{j=1}^{N_{g}} P\left( i, j \right)$ be the marginal column probabilities, $\mu_{x}$ be the mean gray level intensity of $p_{x}$and defined as $\mu_{x}=\sum_{i=1}^{N_{s}} p_{x}\left( i \right)i$, $\mu_{y}$ be the mean gray level intensity of $p_{y}$and defined as $\mu_{y}=\sum_{j=1}^{N_{s}} p_{y}\left( j \right)j$, $\sigma_{x}$ be the standard deviation of $p_{x}$, $\sigma_{y}$ be the standard deviation of $p_{y}$, $p_{x+y}\left( k \right)=\sum_{i=1}^{N_{g}} \sum_{j=1}^{N_{g}} p\left( i,j \right)$, where $i+j=k, and k=2,3,\cdots,2N_{g}$, $p_{x-y}\left( k \right)=\sum_{i=1}^{N_{g}} \sum_{j=1}^{N_{g}} p\left( i,j \right)$, where $\left| i-j \right|=k,$and $k=0,1,\cdots,N_{g}-1$, HX = $-\sum_{i=1}^{N_{g}} p_{x}(i)\log_{2} \left( p_{x}\left( i \right)+\epsilon\right)$ be the entropy of $p_{x}$,

HY = $-\sum_{j=1}^{N_{g}} p_{y}(j)\log_{2} \left( p_{y}\left( j \right)+\epsilon\right)$ be the entropy of $p_{y}$,

HXY = $-\sum_{i=1}^{N_{g}} \sum_{j=1}^{N_{g}} p\left( i,j \right)\log_{2} \left( p\left( i,j \right)+\epsilon\right)$ be the entropy of $p\left( i,j \right),$

HXY1 = $-\sum_{i=1}^{N_{g}} \sum_{j=1}^{N_{g}} p\left( i,j \right)\log_{2} \left( {p_{x}\left( i \right)p}_{y}\left( j \right)+\epsilon\right)$, and

HXY2 = $-\sum_{i=1}^{N_{g}} \sum_{j=1}^{N_{g}} {p_{x}\left( i \right)p}_{y}\left( j \right)\log_{2} \left( {p_{x}\left( i \right)p}_{y}\left( j \right)+\epsilon\right)$.

| **Autocorrelation** | $\sum_{i=1}^{N_{g}} \sum_{j=1}^{N_{g}} p\left( i,j \right)ij$ | **Joint average** | $\sum_{i=1}^{N_{g}} \sum_{j=1}^{N_{g}} p\left( i,j \right)i$ |
| --- | --- | --- | --- |
| **Cluster prominence** | $\sum_{i=1}^{N_{g}} \sum_{j=1}^{N_{g}} \left( i+j-\mu_{x}-\mu_{y} \right)^{4}p\left( i,j \right)$ | **Inverse difference (ID)** | $\sum_{k=0}^{N_{g}-1} \frac{p_{x-y}\left( k \right)}{1+k}$ |
| **Cluster shade** | $\sum_{i=1}^{N_{g}} \sum_{j=1}^{N_{g}} \left( i+j-\mu_{x}-\mu_{y} \right)^{3}p\left( i,j \right)$ | **Inverse difference normalized (IDN)** | $\sum_{k=0}^{N_{g}-1} \frac{p_{x-y}\left( k \right)}{1+\left( \frac{k}{N_{g}} \right)}$ |
| **Cluster tendency** | $\sum_{i=1}^{N_{g}} \sum_{j=1}^{N_{g}} \left( i+j-\mu_{x}-\mu_{y} \right)^{2}p\left( i,j \right)$ | **Inverse difference moment (IDM)** | $\sum_{k=0}^{N_{g}-1} \frac{p_{x-y}\left( k \right)}{1+k^{2}}$ |
| **Contrast** | $\sum_{i=1}^{N_{g}} \sum_{j=1}^{N_{g}} \left( i-j \right)^{2}p\left( i,j \right)$ | **Inverse difference moment normalized (IDMN)** | $\sum_{k=0}^{N_{g}-1} \frac{p_{x-y}\left( k \right)}{1+\left( \frac{k^{2}}{{N_{g}}^{2}} \right)}$ |
| **Correlation** | $\frac{\sum_{i=1}^{N_{g}} \sum_{j=1}^{N_{g}} p\left( i,j \right)ij-\mu_{x}\mu_{y}}{\sigma_{x}\left( i \right)\sigma_{y}(i)}$ | **Inverse variance** | $\sum_{k=1}^{N_{g}-1} \frac{p_{x-y}\left( k \right)}{k^{2}}$ |
| **Difference average** | $\sum_{k=0}^{N_{g}-1} kp_{x-y}\left( k \right)$ | **Joint energy** | $\sum_{i=1}^{N_{g}} \sum_{j=1}^{N_{g}} \left( p(i,j) \right)^{2}$ |
| **Difference entropy** | $\sum_{k=0}^{N_{g}-1} {p_{x-y}\left( k \right)log}_{2}\left( p_{x-y}\left( k \right)+\epsilon\right)$ | **Joint entropy** | $-\sum_{i=1}^{N_{g}} \sum_{j=1}^{N_{g}} p(i,j)\log_{2} \left( p(i,j)+\epsilon\right)$ |
| **Difference variance** | $\sum_{k=0}^{N_{g}-1} \left( k-DA \right)^{2}p_{x-y}\left( k \right)$ | **Maximum probability** | $max(p\left( i,j \right))$ |
| **Sum of squares** | $\sum_{i=1}^{N_{g}} \sum_{j=1}^{N_{g}} \left( i-\mu_{x} \right)^{2}p(i,j)$ | **Maximal correlation coefficient (MCC)** | $\sqrt{\begin{aligned} second largest \\ eigenvalue of Q \end{aligned}}$  $Q\left( i,j \right)=\sum_{k=0}^{N_{g}} \frac{p\left( i,k \right)p(j,k)}{p_{x}(i)p_{y}(k)}$ |
| **Sum average** | $\sum_{k=2}^{2N_{g}} p_{x+y}\left( k \right)k$ | **Informational measure of correlation (IMC) 1** | $\frac{HXY-HXY1}{max\left\{ HX,HY \right\}}$ |
| **Sum entropy** | $\sum_{k=2}^{2N_{g}} p_{x+y}\left( k \right)\log_{2} p_{x+y}\left( k \right)+\epsilon)$ | **Informational measure of correlation (IMC) 2** | $\sqrt{1-e^{-2(HXY2-HXY)}}$ |

***3-2. Gray level run length matrix (GLRLM) features***

Gray level run length matrix (GLRLM) quantifies gray level runs, which are defined as the length in number of pixels, of consecutive pixels that had the same gray level value. In a gray level run length matrix $P(i,j|\theta)$, the ${(i,j)}^{th}$element described the number of runs with gray level $i$ and length $i$ occurred in the image (ROI) along angle $\theta$.

Let $N_{g}$ be the number of discreet intensity values in the image, $N_{s}$ be the number of discreet zone sizes in the image, $N_{r}$ be the number of discreet run lengths in the image, $N_{p}$ be the number of voxels in the image,$N_{r}\left( \theta\right)$ be the number of runs in the image along angle $\theta$, which is equal to $\sum_{i=1}^{N_{g}} \sum_{j=1}^{N_{g}} P(i,j|\theta)$ and 1$\leq N_{r}\left( \theta\right)\leq N_{p}$, $P\left( i,j|\theta\right)$ be the run length matrix for an arbitrary direction $\theta$, and $p(i,j|\theta)$ be the normalized run length matrix, defined as $p\left( i,j|\theta\right)=\frac{P(i,j|\theta)}{N_{r}\left( \theta\right)}$.

| **Short run emphasis (SRE)** | $\frac{\sum_{i=1}^{N_{g}} \sum_{j=1}^{N_{r}} \frac{P(i,j\vert\theta)}{j^{2}}}{N_{r}\left( \theta\right)}$ | **Run variance (RV)** | $\sum_{i=1}^{N_{g}} \sum_{j=1}^{N_{r}} {p(i,j\vert\theta)\left( j-\mu\right)}^{2}$  $\mu=\sum_{i=1}^{N_{g}} \sum_{j=1}^{N_{r}} p\left( i,j\vert\theta\right)j$ |
| --- | --- | --- | --- |
| **Long run emphasis (LRE)** | $\frac{\sum_{i=1}^{N_{g}} \sum_{j=1}^{N_{r}} P(i,j\vert\theta)j^{2}}{N_{r}\left( \theta\right)}$ | **Run entropy (RE)** | $-\sum_{i=1}^{N_{g}} \sum_{j=1}^{N_{r}} p\left( i,j\vert\theta\right)\log_{2}$  $\left( p\left( i,j\vert\theta\right)+ \epsilon\right)$ |
| **Gray level non-uniformity (GLN)** | $\frac{\sum_{i=1}^{N_{g}} \left( \sum_{j=1}^{N_{r}} P(i,j\vert\theta) \right)^{2}}{N_{r}\left( \theta\right)}$ | **Low gray level run emphasis (LGLRE)** | $\frac{\sum_{i=1}^{N_{g}} \sum_{j=1}^{N_{r}} \frac{P(i,j\vert\theta)}{i^{2}}}{N_{r}\left( \theta\right)}$ |
| **Gray level non-uniformity normalized (GLNN)** | $\frac{\sum_{i=1}^{N_{g}} \left( \sum_{j=1}^{N_{r}} P(i,j\vert\theta) \right)^{2}}{{N_{r}\left( \theta\right)}^{2}}$ | **High gray level run emphasis (HGLRE)** | $\frac{\sum_{i=1}^{N_{g}} \sum_{j=1}^{N_{r}} P(i,j\vert\theta)i^{2}}{N_{r}\left( \theta\right)}$ |
| **Run length non-uniformity (RLN)** | $\frac{\sum_{j=1}^{N_{r}} \left( \sum_{i=1}^{N_{g}} P(i,j\vert\theta) \right)^{2}}{N_{r}\left( \theta\right)}$ | **Short run low gray level emphasis (SRLGLE)** | $\frac{\sum_{i=1}^{N_{g}} \sum_{j=1}^{N_{r}} \frac{P(i,j\vert\theta)}{i^{2}j^{2}}}{N_{r}\left( \theta\right)}$ |
| **Run length non-uniformity normalized (RLNN)** | $\frac{\sum_{j=1}^{N_{r}} \left( \sum_{i=1}^{N_{g}} P(i,j\vert\theta) \right)^{2}}{{N_{r}\left( \theta\right)}^{2}}$ | **Short run high gray level emphasis (SRHGLE)** | $\frac{\sum_{i=1}^{N_{g}} \sum_{j=1}^{N_{r}} \frac{P(i,j\vert\theta)i^{2}}{j^{2}}}{N_{z}}$ |
| **Run percentage (RP)** | $\frac{N_{r}\left( \theta\right)}{N_{p}}$ | **Long run low gray level emphasis (LRLGRE)** | $\frac{\sum_{i=1}^{N_{g}} \sum_{j=1}^{N_{r}} \frac{P(i,j\vert\theta)j^{2}}{i^{2}}}{N_{r}\left( \theta\right)}$ |
| **Gray level variance (GLV)** | $\sum_{i=1}^{N_{g}} \sum_{j=1}^{N_{r}} {p\left( i,j\vert\theta\right)\left( i-\mu\right)}^{2}$  $\mu=\sum_{i=1}^{N_{g}} \sum_{j=1}^{N_{r}} p\left( i,j\vert\theta\right)i$ | **Long run high gray level emphasis (LRHGLE)** | $\frac{\sum_{i=1}^{N_{g}} \sum_{j=1}^{N_{r}} P(i,j\vert\theta)i^{2}j^{2}}{N_{r}\left( \theta\right)}$ |

***3-3. Gray level size zone matrix (GLSZM) features***

Gray level size zone matrix (GLSZM) quantifies gray level zones in an image. A gray level zone was defined as the number of connected voxels that shared the same gray level intensity. A voxel was considered connected if the distance was 1 according to the infinity norm (26-connected region in a 3D and 8-connected region in 2D). In a gray level size zone matrix $p(i,j)$, the ${(i,j)}^{th}$ element equaled the number of zones with gray level $i$ and size $j$ appeared in image. Contrary to the GLCM and GLRLM, the GLSZM was rotation independent, with only one matrix calculated for all directions in the ROI.

Let $N_{g}$ be the number of discreet intensity values in the image, $N_{s}$ be the number of discreet zone sizes in the image, $N_{p}$ be the number of voxels in the image, $N_{z}$ be the number of zones in the ROI, which is equal to $\sum_{i=1}^{N_{g}} \sum_{j=1}^{N_{g}} P(i,j)$ and 1$\leq N_{z}\leq N_{p}$, $P\left( i,j \right)$ be the size zone matrix, and $p(i,j)$ be the normalized size zone matrix, defined as $p\left( i,j \right)=\frac{P(i,j)}{N_{z}}$.

| **Small area emphasis (SAE)** | $\frac{\sum_{i=1}^{N_{g}} \sum_{j=1}^{N_{s}} \frac{P(i,j)}{j^{2}}}{N_{z}}$ | **Zone variance (ZV)** | $\sum_{i=1}^{N_{g}} \sum_{j=1}^{N_{g}} {p(i,j)\left( j-\mu\right)}^{2}$  $\mu=\sum_{i=1}^{N_{g}} \sum_{j=1}^{N_{g}} p\left( i,j \right)j$ |
| --- | --- | --- | --- |
| **Large area emphasis (LAE)** | $\frac{\sum_{i=1}^{N_{g}} \sum_{j=1}^{N_{s}} P(i,j)j^{2}}{N_{z}}$ | **Zone entropy (ZE)** | $-\sum_{i=1}^{N_{g}} \sum_{j=1}^{N_{s}} p\left( i,j \right)\log_{2}$  $( p\left( i,j \right)+\epsilon)$ |
| **Gray level non-uniformity (GLN)** | $\frac{\sum_{i=1}^{N_{g}} \left( \sum_{j=1}^{N_{s}} P(i,j) \right)^{2}}{N_{z}}$ | **Low gray level zone emphasis (LGLZE)** | $\frac{\sum_{i=1}^{N_{g}} \sum_{j=1}^{N_{s}} \frac{P(i,j)}{i^{2}}}{N_{z}}$ |
| **Gray level non-uniformity normalized (GLNN)** | $\frac{\sum_{i=1}^{N_{g}} \left( \sum_{j=1}^{N_{s}} P(i,j) \right)^{2}}{{N_{z}}^{2}}$ | **High gray level zone emphasis (HGLZE)** | $\frac{\sum_{i=1}^{N_{g}} \sum_{j=1}^{N_{s}} P(i,j)i^{2}}{N_{z}}$ |
| **Size-zone non-uniformity (SZN)** | $\frac{\sum_{j=1}^{N_{s}} \left( \sum_{i=1}^{N_{g}} P(i,j) \right)^{2}}{N_{z}}$ | **Small area low gray level emphasis (SALGLE)** | $\frac{\sum_{i=1}^{N_{g}} \sum_{j=1}^{N_{s}} \frac{P(i,j)}{i^{2}j^{2}}}{N_{z}}$ |
| **Size-zone non-uniformity normalized (SZNN)** | $\frac{\sum_{j=1}^{N_{s}} \left( \sum_{i=1}^{N_{g}} P(i,j) \right)^{2}}{{N_{z}}^{2}}$ | **Small area high gray level emphasis (SAHGLE)** | $\frac{\sum_{i=1}^{N_{g}} \sum_{j=1}^{N_{s}} \frac{P(i,j)i^{2}}{j^{2}}}{N_{z}}$ |
| **Zone percentage (ZP)** | $\frac{N_{z}}{N_{p}}$ | **Large area low gray level emphasis (LALGLE)** | $\frac{\sum_{i=1}^{N_{g}} \sum_{j=1}^{N_{s}} \frac{P(i,j)j^{2}}{i^{2}}}{N_{z}}$ |
| **Gray level variance (GLV)** | $\sum_{i=1}^{N_{g}} \sum_{j=1}^{N_{s}} {p\left( i,j \right)\left( i-\mu\right)}^{2}$  $\mu=\sum_{i=1}^{N_{g}} \sum_{j=1}^{N_{s}} p\left( i,j \right)i$ | **Large area high gray level emphasis (LAHGLE)** | $\frac{\sum_{i=1}^{N_{g}} \sum_{j=1}^{N_{s}} P(i,j)i^{2}j^{2}}{N_{z}}$ |

***3-4. Gray level dependence matrix (GLDM) features***

Gray level dependence matrix (GLDM) quantifies gray level dependencies in an image. A gray level dependency was defined as the number of connected voxels within distance δ that are dependent on the center voxel. A neighboring voxel with gray level $j$ was considered dependent on center voxel with gray level $i$ if |$i-j$|≤α. In a gray level dependence matrix $P(i,j)$, the  ${(i,j)}^{th}$ element described the number of times a voxel with gray level $i$ with $j$ dependent voxels in its neighborhood appears in image.

Let $N_{g}$ be the number of discreet intensity values in the image, $N_{d}$ be the number of discreet dependency sizes in the image, $N_{z}$ be the number of dependency zones in the image, which is equal to $\sum_{i=1}^{N_{g}} \sum_{j=1}^{N_{d}} P(i,j)$, $P\left( i,j \right)$ be the dependence matrix, and $p(i,j)$ be the normalized dependence matrix, defined as $p\left( i,j \right)=\frac{P(i,j)}{N_{z}}$.

| **Small dependence emphasis (SDE)** | $\frac{\sum_{i=1}^{N_{g}} \sum_{j=1}^{N_{d}} \frac{P(i,j)}{i^{2}}}{N_{z}}$ | **Dependence variance (DV)** | $\sum_{i=1}^{N_{g}} \sum_{j=1}^{N_{d}} {p(i,j)\left( j-\mu\right)}^{2}$  $\mu=\sum_{i=1}^{N_{g}} \sum_{j=1}^{N_{d}} p\left( i,j \right)j$ |
| --- | --- | --- | --- |
| **Large dependence emphasis (LDE)** | $\frac{\sum_{i=1}^{N_{g}} \sum_{j=1}^{N_{d}} P(i,j)j^{2}}{N_{z}}$ | **Low gray level emphasis (LGLE)** | $\frac{\sum_{i=1}^{N_{g}} \sum_{j=1}^{N_{d}} \frac{P(i,j)}{i^{2}}}{N_{z}}$ |
| **Gray level non-uniformity (GLN)** | $\frac{\sum_{i=1}^{N_{g}} \left( \sum_{j=1}^{N_{d}} P(i,j) \right)^{2}}{N_{z}}$ | **High gray level emphasis (HGLE)** | $\frac{\sum_{i=1}^{N_{g}} \sum_{j=1}^{N_{d}} P(i,j)i^{2}}{N_{z}}$ |
| **Dependence non-uniformity (DN)** | $\frac{\sum_{j=1}^{N_{d}} \left( \sum_{i=1}^{N_{g}} P(i,j) \right)^{2}}{N_{z}}$ | **Small dependence low gray level emphasis (SDLGLE)** | $\frac{\sum_{i=1}^{N_{g}} \sum_{j=1}^{N_{d}} \frac{P(i,j)}{i^{2}j^{2}}}{N_{z}}$ |
| **Dependence non-uniformity normalized (DNN)** | $\frac{\sum_{j=1}^{N_{d}} \left( \sum_{i=1}^{N_{g}} P(i,j) \right)^{2}}{{N_{z}}^{2}}$ | **Small dependence high gray level emphasis (SDHGLE)** | The joint distribution of small dependence with higher gray-level values. |
| **Dependence entropy (DE)** | $-\sum_{i=1}^{N_{g}} \sum_{j=1}^{N_{d}} p\left( i,j \right)\log_{2}( p\left( i,j \right)+\epsilon$) | **Large dependence high gray level emphasis (LDHGLE)** | $\frac{\sum_{i=1}^{N_{g}} \sum_{j=1}^{N_{d}} P(i,j)i^{2}j^{2}}{N_{z}}$ |
| **Gray level variance (GLV)** | $\sum_{i=1}^{N_{g}} \sum_{j=1}^{N_{d}} {p\left( i,j \right)\left( i-\mu\right)}^{2}$  $\mu=\sum_{i=1}^{N_{g}} \sum_{j=1}^{N_{d}} p\left( i,j \right)i$ | **Large dependence low gray level emphasis (LDLGLE)** | $\frac{\sum_{i=1}^{N_{g}} \sum_{j=1}^{N_{d}} \frac{P(i,j)j^{2}}{i^{2}}}{N_{z}}$ |

***3-5. Neighboring gray tone difference matrix (NGTDM) features***

Neighboring gray tone difference matrix (NGTDM) quantifies the difference between a gray value and the average gray value of its neighbors within distance δ. The sum of absolute differences for gray level $i$ was stored in the matrix.

Let $X_{gl}$ be a set of segmented voxels and $x_{gl}(j_{x}$, $j_{y}, j_{z})\in X_{gl}$ be the gray level of a voxel at postion $(j_{x}$,$j_{y}, j_{z})$, then the average gray level of the neigborhood is:

$\bar{A}_{i}=\bar{A}(j_{x}$,$j_{y}, j_{z})=\frac{1}{W}\sum_{k_{x}=-\delta}^{\delta} \sum_{k_{y}=-\delta}^{\delta} \sum_{k_{z}=-\delta}^{\delta} x_{gl}(j_{x}+k_{x}, j_{y}+k_{y}, j_{z}+k_{z}),$

where $(k_{x}$,$k, k_{z})\neq(0, 0, 0)$ and $x_{gl}(j_{x}+k_{x}, j_{y}+k_{y}, j_{z}+k_{z})\in X_{gl}$. *W* is the number of voxels in the neighborhood that are also in $X_{gl}$.

Let $n_{i}$ be the number of voxels in $X_{gl}$ with gray level $i$, $N_{v,p}$ be the total number of voxels in $X_{gl}$ and equal to $\sum n_{i}$(i.e., the number of voxels with a valid region; at least 1 neighbor). $N_{v,p}\leq N_{p}$, where $N_{p}$ is the total number of voxels in the ROI, $p_{i}$ be the gray level probability and equal to $n_{i}$/$N_{v}$, $s_{i}=\left\{ \begin{matrix} \sum^{n_{i}} \left| i-\bar{A}_{i} \right| & for & n_{i}\neq0 \\ 0 & for & n_{i}=0 \end{matrix} \right.$ be the sum of absolute differences for gray level $i$, $N_{g}$ be the number of discreet gray levels, and $N_{g,p}$be the number of gray levels where $p_{i}\neq0$.

| **Coarseness** | $\frac{1}{\sum_{i=1}^{N_{g}} p_{i}s_{i}}$ | | **Complexity** | $\frac{1}{N_{v,p}}\sum_{i=1}^{N_{g}} \sum_{j=1}^{N_{g}} \left\vert i-j \right\vert\frac{p_{i}s_{i}+p_{j}s_{j}}{p_{i}{+p}_{i}}$,  where $p_{i}\neq0, p_{j}\neq0$ |
| --- | --- | --- | --- | --- |
| **Busyness** | $\frac{\sum_{i=1}^{N_{g}} p_{i}s_{i}}{\sum_{i=1}^{N_{g}} \sum_{j=1}^{N_{g}} \left\vert{ip}_{i}-jp_{j} \right\vert}$,  where $p_{i}\neq0, p_{j}\neq0$ | | **Strength** | $\frac{\sum_{i=1}^{N_{g}} \sum_{j=1}^{N_{g}} (p_{i}+p_{j}){(i-j)}^{2}}{\sum_{i=1}^{N_{g}} s_{i}}$),  where $p_{i}\neq0, p_{j}\neq0$ |
| **Contrast** | | $\left( \frac{1}{N_{g,p}(N_{g,p}-1)}\sum_{i=1}^{N_{g}} \sum_{j=1}^{N_{g}} p_{i}p_{j}{(i-j)}^{2} \right)\left( \frac{1}{N_{v,p}}\sum_{i=1}^{N_{g}} s_{i} \right)$, where $p_{i}\neq0, p_{j}\neq0$ | | |

**S2. Definition of F1 score**

The F1 score is the harmonic mean of positive predictive value (also known as precision) and sensitivity (also known as recall) and can range between 1 (perfect classification) and 0. It is calculated as follows:

$$F1 score=2 \times\frac{PPV \times sensitivity}{PPV+sensitivity}$$

F1 score is known to be a useful metric to see the performance of a classifier in an imbalanced dataset ^2^.

**S3. Definition of Fréchet Inception distance (FID)**

Fréchet Inception distance (FID) embeds a set of generated samples into a feature space given by a specific layer of Inception Net (or any convolutional neural network) ^3^. Viewing the embedding layer as a continuous mutivariate normal distribution, the FID is given by,

FID= ∣∣μX ​− μY​∣∣2 – Tr (∑X​ + ∑Y​ − 2∑X​∑Y​​)

Where X and Y are the real and fake embeddings (activation from the Inception model) assumed to be two multivariate normal distributions. (μX, ∑X) and (μY, ​∑Y) are the mean and covariance of the real data and model distributions, respectively. Lower FID means smaller distances between synthetic and real data distributions.

**Supplementary Figure 1. Patient flowchart in the (a) institutional and (b) external validation datasets.**

**Supplementary Figure 2. MI scores of the selected radiomic features.** Higher MI scores mean higher dependency between variables.

MI = mutual information

**Supplementary Figure 3.** **Bar graph showing the FID score between the institutional training set and original external validation set, and the FID score between the institutional training set and external validation set after style transfer.** The FID score decreased after style transfer of the external validation (Decrease to 52.2%).

**Supplementary Figure 4. t**-**SNE visualizations of radiomic feature vectors in the institutional training set and external validation set before and after style transfer with CycleGAN.** Each point represents a feature vector for one patient. The institutional training set and original external validation set before style transfer show a different distribution (a), whereas after style transfer, the external validation set shows nearly identical distribution to the institutional training set (b).

CycleGAN = Cycle-Consistent Adversarial Networks, t-SNE = t-distributed stochastic neighbor embedding.

**Supplementary Figure 5. Examples of the density plots showing the distribution of the selected radiomic features.** (a) T1C_GLRLM_LongRunHighGrayLevelEmphasis and (b) T1C_Firstorder_Kurtosis for low-grade and high-grade meningiomas within each dataset. The distributions of radiomic feaetures of the external validation set became more similar to the institutional training set after applying CycleGAN.

**Supplementary Table 1. Comparison of MRI parameters of patients in the training cohort (from our institution) and those in the external validation cohort.**

|  | Institutional set | External validation set | |
| --- | --- | --- | --- |
|  | Severance Hospital | Ewha Mokdong University Hospital | |
| MRI vendor | Philips Achieva/Ingenia | Siemens Avanto | Philips Achieva |
| Tesla | 3.0 | 1.5 | 3.0 |
| T2 |  |  |  |
| Repetition Time (ms) | 3000–9000 | 5000-5800 | 3000 |
| Echo Time (ms) | 80–120 | 100-120 | 80 |
| Pixel size (mm) | 0.9–1 | 0.35-0.44 | 0.43-0.46 |
| Section thickness (mm) | 3–5 | 5 | 5 |
| T1C |  |  |  |
| Repetition Time (ms) | 6.3-8.3 | 550-590 | 1800-2000 |
| Echo Time (ms) | 2.9-4 | 11-12 | 20-22 |
| Pixel size (mm) | 0.9–1 | 0.50-0.58 | 0.60-0.67 |
| Section thickness (mm) | 1-2 | 3-5 | 2-5 |

T1C = postcontrast T1-weighted image; T2 = T2-weighted image

**Supplementary Table 2. List of extracted radiomics features.**

| Feature category | Feature list |
| --- | --- |
| Shape (n = 14) | Volume, surface area, surface area to volume ratio, sphericity, maximum 3D diameter, maximum 2D diameter (column), maximum 2D diameter (row), maximum 2D diameter (slice), major axis, minor axis, least axis, elongation, flatness, mesh volume |
| First order statistics (n = 18) | Energy, total energy, entropy, minimum, 10^th^ percentile, 90^th^ percentile, maximum, mean, median, interquartile range, range, mean absolute deviation, robust mean absolute deviation, root mean squared, skewness, kurtosis, variance, uniformity |
| GLCM (n = 24) | Autocorrelation, cluster prominence, cluster shade, cluster tendency, contrast, correlation, difference average, difference entropy, difference variance, inverse difference, inverse difference moment, inverse difference moment normalized, inverse difference normalized, informal measure of correlation 1, informal measure of correlation 2, inverse variance, joint average, joint energy, joint entropy, maximal correlation coefficient, maximum probability, sum average, sum entropy, sum of squares |
| GLRLM (n = 16) | Short-run emphasis, long-run emphasis, gray level nonuniformity, gray level nonuniformity normalized, run-length nonuniformity, run-length nonuniformity normalized, run percentage, gray level variance, run variance, run entropy, low gray level run emphasis, high gray level run emphasis, short-run low gray level emphasis, short-run high gray level emphasis, long-run low gray level emphasis, long-run high-gray level emphasis |
| GLSZM (n = 16) | Small area emphasis, large area emphasis, gray level non-uniformity, gray level non-uniformity normalized, size-zone non-uniformity, size-zone non-uniformity normalized, zone percentage, gray level variance, zone variance, zone entropy, low gray level zone emphasis, high gray level zone emphasis, small area low gray level emphasis, small area high gray level emphasis, large area low gray level emphasis, large area high gray level emphasis |
| GLDM (n = 14) | Small dependence emphasis, large dependence emphasis, gray level non-uniformity, dependence non-uniformity, dependence non-uniformity normalized, gray level variance, dependence variance, dependence entropy, low gray level emphasis, high gray level emphasis, small dependence low gray level emphasis, small dependence high gray level emphasis, large dependence low gray level emphasis, large dependence high gray level emphasis, |
| NTGDM (n = 5) | Coarseness, complexity, strength, contrast, busyness |

GLCM = gray level co-occurrence matrix, GLDM = gray level dependence matrix, GLRLM = gray level run-length matrix, GLSZM = gray level size zone matrix, NTGDM = neighboring gray tone difference matrix

**Supplementary Table 3. Detailed CycleGAN Architecture**

| **Generator Network Architecture** | | | | | | | | | | | | |
| --- | --- | --- | --- | --- | --- | --- | --- | --- | --- | --- | --- | --- |
| Phase | | Filter Number | | Filter Size | Layer Type | | Stride | | Normalization | | Activation | Representation Size |
| Encoder | | 64 | | 7x7 | Convolution | | 1 | | InstanceNorm | | ReLU | k |
|  |  | 128 | | 3x3 | Convolution | | 2 | | InstanceNorm | | ReLU | k/2 |
|  |  | 256 | | 3x3 | Convolution | | 2 | | InstanceNorm | | ReLU | k/4 |
| Transformer | | 256 | | 3x3 | Residual Block | | 1 | | InstanceNorm | | ReLU | k/4 |
|  |  | 256 | | 3x3 | Residual Block | | 1 | | InstanceNorm | | ReLU | k/4 |
|  |  | 256 | | 3x3 | Residual Block | | 1 | | InstanceNorm | | ReLU | k/4 |
|  |  | 256 | | 3x3 | Residual Block | | 1 | | InstanceNorm | | ReLU | k/4 |
|  |  | 256 | | 3x3 | Residual Block | | 1 | | InstanceNorm | | ReLU | k/4 |
|  |  | 256 | | 3x3 | Residual Block | | 1 | | InstanceNorm | | ReLU | k/4 |
| Decoder | | 128 | | 3x3 | Transpose | | 1/2 | | InstanceNorm | | ReLU | k/2 |
|  |  | 64 | | 3x3 | Transpose | | 1/2 | | InstanceNorm | | ReLU | k |
|  |  | 3 | | 7x7 | Transpose | | 1 | | InstanceNorm | | ReLU | k |
| **Discriminator Network Architecture** | | | | | | | | | | | | |
| Filter Number | Filter Size | | Layer Type | | | Stride | | Normalization | | Activation | | |
| 64 | 4x4 | | Convolution | | | 2 | | - | | LeakyReLU (slope=0.2) | | |
| 128 | 4x4 | | Convolution | | | 2 | | InstanceNorm | | LeakyReLU (slope=0.2) | | |
| 256 | 4x4 | | Convolution | | | 2 | | InstanceNorm | | LeakyReLU (slope=0.2) | | |
| 512 | 4x4 | | Convolution | | | 2 | | InstanceNorm | | LeakyReLU (slope=0.2) | | |
| 1 | 4x4 | | Convolution | | | 1 | | - | | Sigmoid | | |

CycleGAN = Cycle-Consistent Adversarial Networks; ReLU = Rectified Linear Unit

**Supplementary Table 4. List of significant multiparametric radiomic features to differentiate meningioma grade using MI.**

| Image sequence | Feature category | Feature name |
| --- | --- | --- |
| T2 | First order | Uniformity |
| T2 | GLCM | Joint energy |
| T2 | GLCM | Informational measure of correlation 2 |
| T2 | GLCM | Sum entropy |
| T2 | GLRLM | Long run low gray level emphasis |
| T2 | GLRLM | Gray level non-uniformity normalized |
| T2 | GLSZM | Gray level non-uniformity |
| T2 | GLSZM | Small area low gray level emphasis |
| T2 | GLDM | Gray level non-uniformity |
| T2 | GLDM | Low gray level emphasis |
| T1C | Shape | Surface area |
| T1C | Shape | Voxel volume |
| T2C | Shape | Maximum 3D diameter |
| T3C | Shape | Maximum 2D diameter (column) |
| T4C | Shape | Major axis length |
| T1C | Shape | Minor axis length |
| T1C | First order | Kurtosis |
| T1C | GLCM | Joint energy |
| T1C | GLRLM | Run length non-uniformity |
| T1C | GLRLM | Long run high gray level emphasis |
| T1C | GLRLM | Gray level non-uniformity |
| T1C | GLSZM | Gray level non-uniformity |
| T1C | GLSZM | Size zone non-uniformity |
| T1C | GLDM | Gray level non-uniformity |
| T1C | GLDM | Dependence variance |
| T1C | GLDM | Dependence non-uniformity normalized |
| T1C | GLDM | Small dependence emphasis |

GLCM = gray level co-occurrence matrix; GLDM = gray level dependence matrix; GLRLM = gray level run-length matrix; GLSZM = gray level size zone matrix; T1C = postcontrast T1-weighted image; T2 = T2-weighted image

**References**

1 van Griethuysen, J. J. M. *et al.* Computational Radiomics System to Decode the Radiographic Phenotype. *Cancer research* **77**, e104-e107, doi:10.1158/0008-5472.Can-17-0339 (2017).

2 England, J. R. & Cheng, P. M. Artificial Intelligence for Medical Image Analysis: A Guide for Authors and Reviewers. *AJR Am J Roentgenol* **212**, 513-519, doi:10.2214/ajr.18.20490 (2019).

3 Borji, A. Pros and cons of gan evaluation measures. *Computer Vision and Image Understanding* **179**, 41-65 (2019).
