# Supplementary figures and images for: Cycle-consistent adversarial networks improves generalizability of radiomics model in grading meningiomas on external validation
Source: Sci Rep. 2022 Apr 29;12:7042. doi: 10.1038/s41598-022-10956-9 (PMC9055063; doi:10.1038/s41598-022-10956-9)

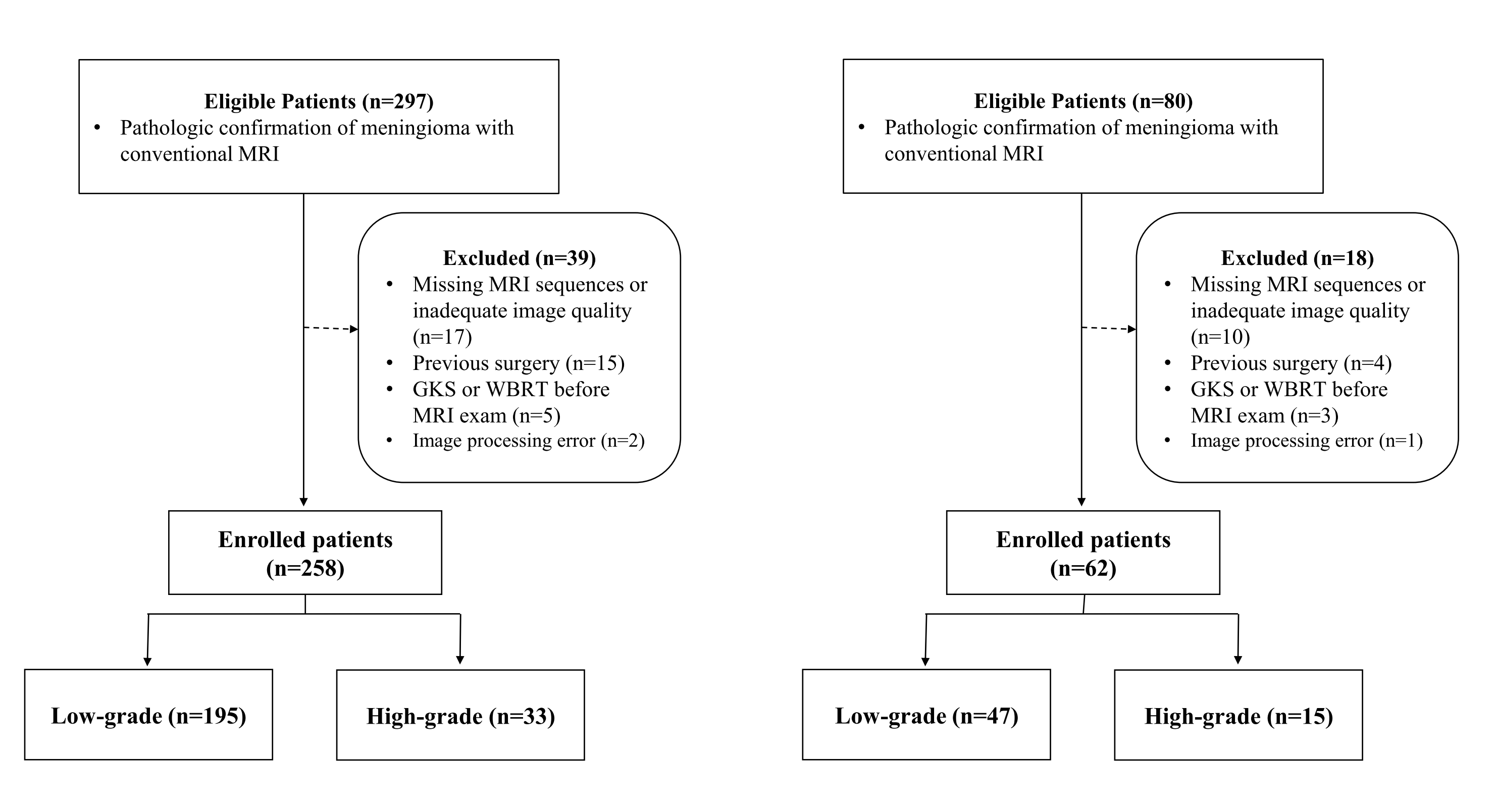

Supplement: Supplementary file 2 — Supplementary Information 2. [file 41598_2022_10956_MOESM2_ESM.tif]

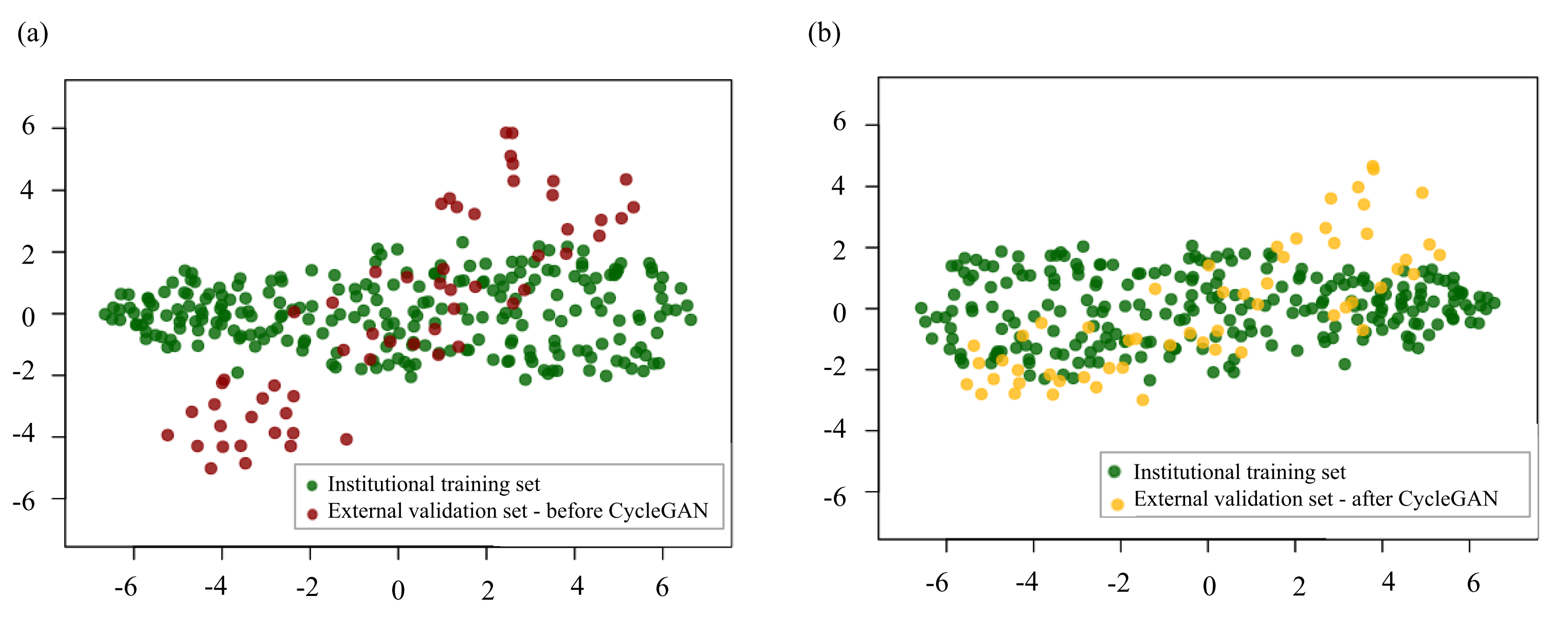

Supplement: Supplementary file 5 — Supplementary Information 5. [file 41598_2022_10956_MOESM5_ESM.tif]

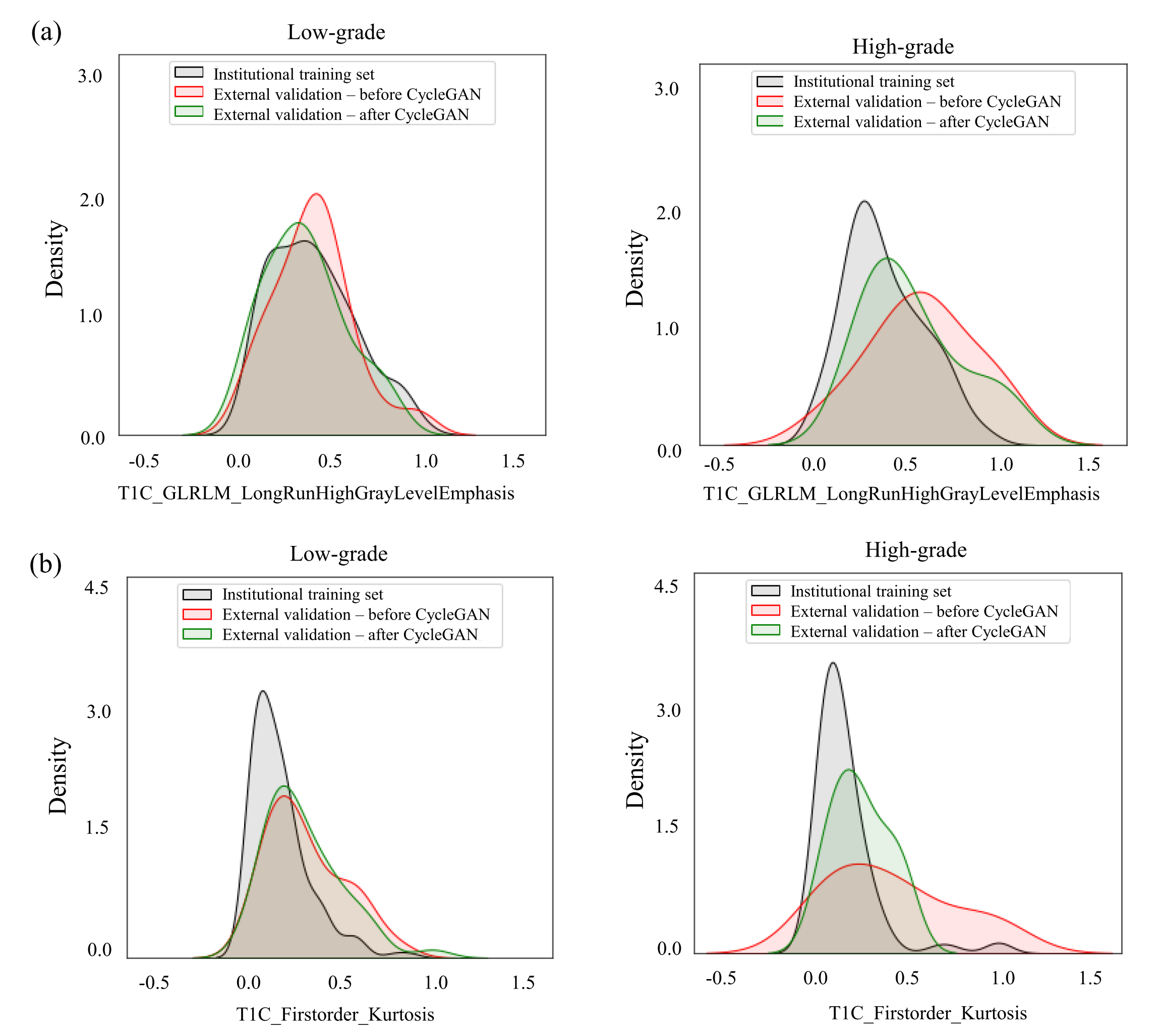

Supplement: Supplementary file 6 — Supplementary Information 6. [file 41598_2022_10956_MOESM6_ESM.tif]
